# Are Noroviruses Emerging?

**DOI:** 10.3201/eid1105.041090

**Published:** 2005-05

**Authors:** Marc-Alain Widdowson, Stephan S. Monroe, Roger I. Glass

**Affiliations:** * Centers for Disease Control and Prevention, Atlanta, Georgia, USA

**Keywords:** Epidemiology, Norovirus, Communicable Diseases, Emerging

In 1972, noroviruses (previously called "Norwalk-like viruses") were discovered as the first viruses definitively associated with acute gastroenteritis. During the next 2 decades, researchers were unable to develop simple methods to detect these common viruses or to find the etiologic agents of nonbacterial gastroenteritis outbreaks and hospitalizations. Indeed, of >2,500 foodborne outbreaks reported to the Centers for Disease Control and Prevention from 1993 to 1997, <1% were attributed to noroviruses, and 68% were of "unknown etiology" (1). As a result, noroviruses were out of sight and mind and thus relegated to a minor role as agents of gastroenteritis at a time when high-profile outbreaks of *Salmonella* Enteritidis (2) and *Escherichia coli* (3) had focused attention and budgets on preventing foodborne bacterial illnesses.

The development of reverse transcription–polymerase chain reaction in the early 1990s provided the breakthrough needed to facilitate diagnosis of norovirus infection. Today, noroviruses are recognized as the most common cause of infectious gastroenteritis among persons of all ages (4). They are responsible for ≤50% of all foodborne gastroenteritis outbreaks in the United States (5) and are a major contributor to illness in nursing homes (6) and hospitals (7). Noroviruses have been detected in 35% of persons with sporadic gastroenteritis of known cause (8) and in 14% of all children <3 years old hospitalized for gastroenteritis (9). Norovirus infection has put apparently healthy people in intensive care (10) and has been associated with chronic diarrhea among transplant patients (11). In addition, we now know of a myriad of strains of norovirus, which have been classified into 5 genogroups, distinguishable from sapoviruses, a separate genus of human caliciviruses, but also in the *Caliciviridae* family. This diversity represents a dramatic increase from the single calicivirus strain discovered >30 years ago. Moreover, the known host range of noroviruses has expanded: the virus is found in mice (12), cows (13), and pigs (14), and antibodies to bovine strains have been found in humans, which has stimulated speculation about zoonotic transmission (15). However, a fundamental question remains—is the increased detection of norovirus the result of better application of improved diagnostics or does evidence exist that norovirus disease is an emergent problem?

Recent reports have established that norovirus strains can periodically emerge either globally or nationally, displace other strains, and increase disease incidence (16,17). In winter 2002, a new virus variant was attributed to a well-publicized surge of norovirus outbreaks on cruise ships and in nursing homes in the United States (18,19) and in healthcare facilities in Europe (20). Why these strains emerge into prominence is unclear, but they often belong to genogroup II, cluster 4 (Bristol virus). Whether these strains cause different or more severe symptoms than other noroviruses, are more transmissible, or can better evade the host immune response is not known. The periodic emergence of strains is likely to have always been a feature of noroviruses, but we do not know whether norovirus infections are more frequent now than in 1929, when Zahorsky first described "winter vomiting disease" (21). Despite a lack of consistent retrospective data to definitively answer this question, several factors suggest that norovirus disease may actually be more common today.

First, the rates of bacterial foodborne illnesses are declining, in large part because of measures such as improved refrigeration and use of Hazard Analysis and Critical Control Point systems to reduce contamination of food of animal origin (22). Most of these measures, however, will be ineffective against noroviruses, which are resistant to chlorination and freezing, persist in the environment, and require only very low inoculums to infect. Thus, the relative contribution of noroviruses to foodborne disease is likely to be increasing. Second, modern lifestyles make us more vulnerable to norovirus infection than when these viruses were discovered. Since 1972 in the United States, more elderly people live in communal settings, with the number of beds in nursing homes increasing >75% (23). In addition, we now eat more foods that have been handled by a variety of potentially infected people; 46% of household food expenditures is now spent on eating out, compared with 32% in 1972 (24). We also eat more of the foods that are likely to be contaminated with norovirus; consumption of fresh vegetables and fruit has risen >20% in the last 30 years (25), and this produce is often grown in countries where crops are still irrigated with sewage-contaminated water. Finally, more people than ever are traveling and have an increased risk for norovirus infection through exposure to hotels, airplanes, and cruise ships. From 1993 to 1998, for example, the number of cruise ship passengers in the United States increased by 50% (26). Faced with these trends, how should the public health community respond?

First, research on the disease prevalence of noroviruses is only beginning. If noroviruses are an increasingly common cause of infectious gastroenteritis, with some cases resulting in diarrhea-related deaths and hospitalizations, then substantially greater investments are required in their diagnosis. Increased use of diagnostics along with improved surveillance, such as in sentinel sites, will permit identification of new strains and shifts in the epidemiology of norovirus disease. The development of easy-to-use, sensitive assays for use by clinical and public health laboratories should also have a high priority.

Second, we do not know how to stop norovirus transmission. Foods can be contaminated with norovirus either at the source (27) or at the point of service by infected food handlers. Noroviruses can spread by water, direct person-to-person contact, or airborne droplets of vomitus (28), and they can persist in the environment as a source of continuing infection despite efforts at disinfection (29). Recent advances in finding a cell culture system for noroviruses may allow for assessing the efficacy of various disinfectants (30), but only by full epidemiologic investigation of viral gastroenteritis outbreaks and by application of molecular tests will transmission routes be determined, differences in epidemiology between strains be detected, and targeted control measures implemented.

Norovirus infections are common and likely to become more so. Effective prevention strategies must now be designed and implemented.
